# Minimising paper: electronic data capture methods in the 3c study

**DOI:** 10.1186/1745-6215-14-S1-P60

**Published:** 2013-11-29

**Authors:** Richard Haynes

**Affiliations:** 1 University of Oxford, Oxford, UK

## Objective

To establish a cost-effective randomized controlled trial investigating two strategies to improve the long-term function and survival of kidney transplants: The 3C Study (Campath, Calcineurin inhibitor reduction and Chronic allograft nephropathy)

## Methods

In order to recruit at least 800 patients with minimum disruption to their clinical care a bespoke web-based case report form was developed which allows direct data entry by clinicians during routine clinic visits. Access to the web-based system is secure and randomization was conducted online after collection of a small number of data items to allow minimization. Data are verified at entry to avoid missing or impossible data which reduces the requirement for subsequent checks.

In addition to data collected during study visits and annual postal questionnaires, all participants are flagged with national registries which capture all data of interest. The UK Transplant registry provides data to verify baseline characteristics and transplant survival; the UK Renal Registry provides transplant function information; the NHS Information Centre provides cancer and cause-specific mortality data and Hospital Episode Statistics provides information on hospitalisations. Such follow-up provides unbiased, comprehensive follow-up information at very low-cost for the lifetime of the participants which allows the 3C Study to provide uniquely reliable information.

## Results

852 participants were recruited from 18 transplant centres over a 2 year period.

## Conclusion

Innovative use of real-time web-based data entry and long term follow-up through central registries has allowed a large randomized trial in renal transplantation to be established. Similar methods would be applicable to other clinical areas.

